# Investigations towards incorporation of Eu^3+^ and Cm^3+^ during ZrO_2_ crystallization in aqueous solution

**DOI:** 10.1038/s41598-023-39143-0

**Published:** 2023-07-28

**Authors:** Lucas Opitz, René Hübner, Salim Shams Aldin Azzam, Sara E. Gilson, Sarah C. Finkeldei, Nina Huittinen

**Affiliations:** 1 Institute of Resource Ecology, Helmholtz-Zentrum Dresden-Rossendorf, 01328 Dresden, Germany; 2 Department of Chemistry, University of California, Irvine, Irvine, CA 92697 USA; 3 Institute of Ion Beam Physics and Materials Research, Helmholtz-Zentrum Dresden-Rossendorf, 01328 Dresden, Germany; 4 Department of Materials Science and Engineering, University of California, Irvine, Irvine, CA 92697 USA; 5 Department of Chemical and Biomolecular Engineering, University of California, Irvine, Irvine, CA 92697 USA; 6 Institute of Chemistry and Biochemistry, Freie Universität Berlin, 14195 Berlin, Germany

**Keywords:** Geochemistry, Environmental monitoring

## Abstract

Nuclear energy provides a widely applied carbon-reduced energy source. Following operation, the spent nuclear fuel (SNF), containing a mixture of radiotoxic elements such as transuranics, needs to be safely disposed of. Safe storage of SNF in a deep geological repository (DGR) relies on multiple engineered and natural retention barriers to prevent environmental contamination. In this context, zirconia (ZrO_2_) formed on the SNF rod cladding, could be employed as an engineered barrier for immobilization of radionuclides via structural incorporation. This study investigates the incorporation of Eu^3+^ and Cm^3+^, representatives for trivalent transuranics, into zirconia by co-precipitation and crystallization in aqueous solution at 80 °C. Complementary structural and microstructural characterization has been carried out by powder X-ray diffraction (PXRD), spectrum imaging analysis based on energy-dispersive X-ray spectroscopy in scanning transmission electron microscopy mode (STEM-EDXS), and luminescence spectroscopy. The results reveal the association of the dopants with the zirconia particles and elucidate the presence of distinct bulk and superficially incorporated species. Hydrothermal aging for up to 460 days in alkaline media points to great stability of these incorporated species after initial crystallization, with no indication of phase segregation or release of Eu^3+^ and Cm^3+^ over time. These results suggest that zirconia would be a suitable technical retention barrier for mobilized trivalent actinides in a DGR.

## Introduction

Most of the high-level radioactive waste produced by fission of ^235^UO_2_ will eventually be stored in a deep geological repository (DGR). The waste consists primarily of the spent nuclear fuel (SNF), i.e., UO_2_, containing various fission products and transuranics, such as Pu, Np, Am, and Cm. Several of these elements contribute to the long-term radiotoxicity of the SNF, which ultimately will return to the initial level of the natural uranium ore after 10^5^–10^6^ years^1^. For the nuclear waste disposal safety assessment, it is important to understand the chemical behavior of the long-lived, radiotoxic transuranic elements after potential water intrusion into the repository, and the subsequent weathering or corrosion of barrier materials and the SNF matrix itself. Thus, immobilizing reactions, such as the incorporation of transuranics in secondary phases will play a role in determining the fate of these radionuclides in the geosphere^2,3^. One of the first materials that radioactive elements from the waste matrix can interact with is Zircaloy, which is the fuel rod cladding material used in pressurized water reactors. Zirconium-based materials are promising in the context of radiological contamination prevention due to their long-term stability as well as high dopant capacity before undergoing phase separation^4–6^. Already during reactor operation, a passivating zirconia (ZrO_2_) corrosion layer is formed on the cladding surface^7^. In contact with water in a DGR environment, slow dissolution and recrystallization of the ZrO_2_ layer may lead to incorporation of potentially mobile transuranics from the SNF matrix. Zirconia has three naturally occurring polymorphs. At ambient temperature and pressure conditions, the monoclinic (m) phase is the thermodynamically most stable structure. The tetragonal (t) and cubic (c) polymorphs can be stabilized at high temperatures or by incorporation of various aliovalent (e.g. Mg^2+^, Ca^2+^, Fe^3+^, Y^3+^, La^3+^, Nb^5+^)^3,8–14^ and isovalent (e.g. Ce^4+^, Ti^4+^)^15,16^ cations. Incorporation of subvalent cations is accompanied by oxygen vacancy formation in the host structure to retain charge neutrality. These vacancies will thereby reduce the coordination number of the cations in the structure, typically of the Zr^4+^ host. The incorporation of subvalent dopants in ZrO_2_ has been extensively studied using high-temperature (1000–1500 °C) synthesis methods, including co-precipitation or solid-state reaction routes yielding a crystalline, doped ZrO_2_ material. Much less is known about the uptake mechanism of subvalent cations, specifically actinides, into the ZrO_2_ crystal structure during crystallization in aqueous solution in the context of nuclear waste management.

The formation rate of a crystalline zirconia material under aqueous conditions has been shown to depend on the temperature (90–120 °C) and the pH (pH 3–13) of the solution. In very alkaline media a mixture of the tetragonal and monoclinic zirconia structures and a full crystallization of the initially amorphous zirconia precursor has been shown to occur after less than 100 h^17^. Zirconia demonstrates a higher tendency to transform from the tetragonal, often referred to as meta-stable, into the monoclinic phase upon hydrothermal aging, which is influenced mostly by the crystallite size^18^. Shukla and Seal^19^ summarized the effects of various parameters such as surface energy, interfacial energy, etc. on determining the stability of the undoped tetragonal zirconia particles, ultimately resulting in a critical crystallite size upon which the free energy favors the thermodynamically more stable monoclinic phase. Some of the investigated parameters included the surface energy, the interfacial energy, strain energies and multiple other effects that play into the stability range of this phase. The critical crystallite size for the tetragonal structure to be thermodynamically stable was estimated to be in a range between 10 and 33 nm.

Upon incorporation of dopants into the zirconia crystal structure, the high-temperature t + c crystal structures are stabilized during hydrothermal synthesis. The kinetics of the crystallization from the amorphous hydrous precursor, depending on different dopants and concentrations, show slightly faster crystallization rates compared to undoped zirconia^20^. This is likely due to the coexistence of stabilized tetragonal and/or cubic structures in the zirconia material. Low-temperature hydrothermal approaches after successful co-precipitation yield fully incorporated species with no sign of phase segregation^21^.

This study aims to understand the incorporation of trivalent actinides (Cm^3+^) and lanthanides (Eu^3+^) into ZrO_2_ in aqueous solution starting from a hydrous zirconia precursor. A pH of 12 and a temperature of 80 °C are chosen for the aqueous syntheses in the present study as both alkaline conditions and elevated temperatures have been shown to enhance the crystallization rate and conversion of hydrous zirconia to ZrO_2_^17,22^. Moreover, high pH values have been shown to arise in the near-field of a DGR following e.g. canister corrosion and weathering of cementitious backfill and sealing materials^23–25^, while elevated temperatures are expected in the presence of the heat-generating SNF matrix, depending on its age and composition^26,27^. The experiments are designed to study the influence of the dopants, their concentration, and the hydrothermal aging time on the incorporation mechanism. The two studied dopants have been chosen due to their + 3 oxidation state being the most stable one in the aqueous phase and their excellent luminescence properties making it possible to study their coordination environment via luminescence spectroscopy. Powder X-ray diffraction (PXRD) and spectrum imaging analysis based on energy-dispersive X-ray spectroscopy in scanning transmission electron microscopy mode (STEM-EDXS) provide further insights into the phase composition and the spatial distribution of the elements in the ZrO_2_ structure.

## Results

### Powder X-ray diffraction (PXRD)

**Figure 1:**
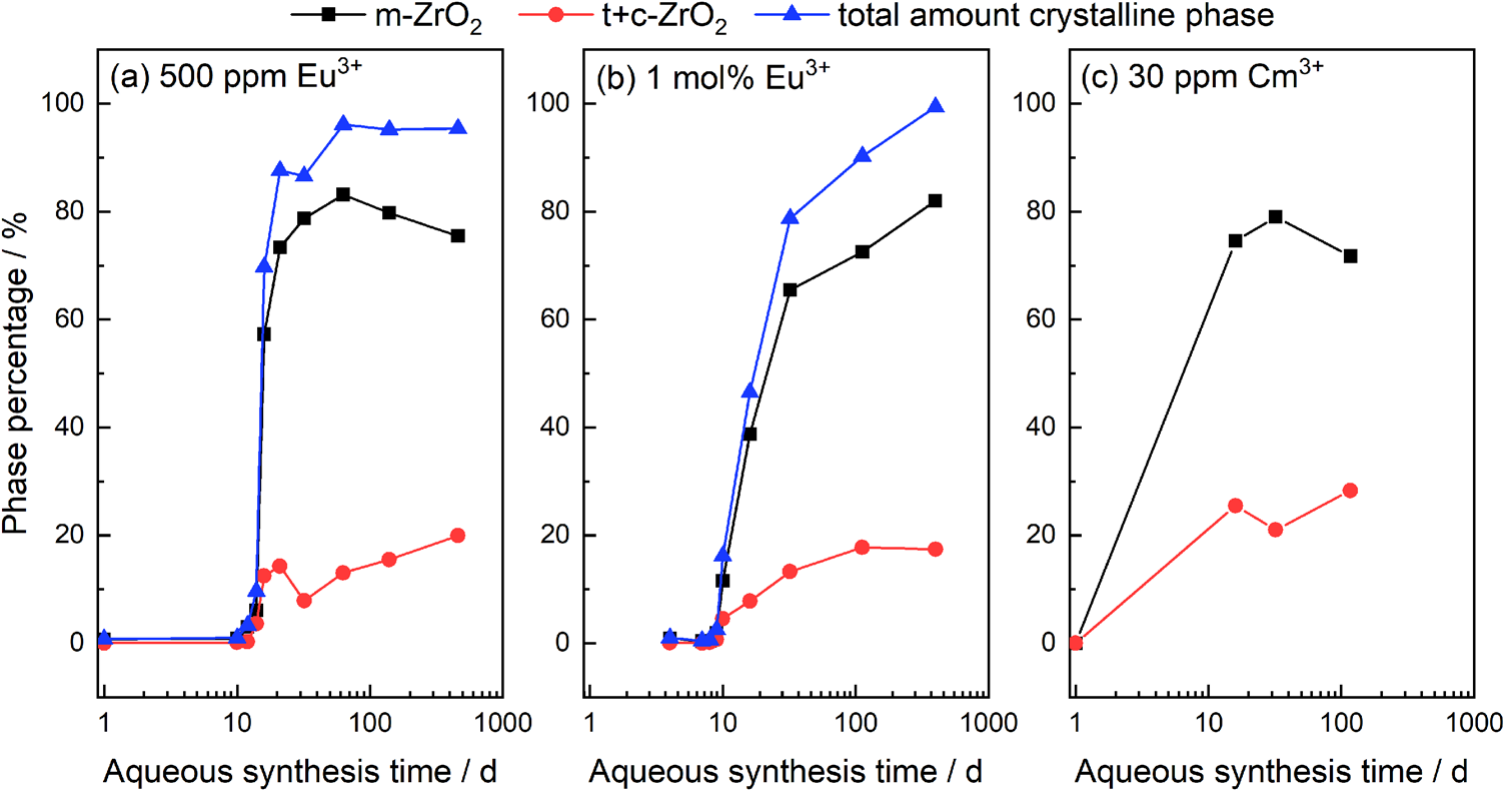
Phase composition of the ZrO_2_ samples containing 500 ppm Eu^3+^ (**a**), 1 mol% Eu^3+^ (**b**), and 30 ppm Cm^3+^ (**c**), with the monoclinic phase depicted in squares (black), the tetragonal and cubic phase in circles (red), and the overall crystallinity of the Eu^3+^-doped samples shown as triangles (blue) determined with Si as internal standard. Synthesis conditions: T = 80 °C, 0.5 M NaCl, pH 12.

The Eu^3+^- and Cm^3+^-doped hydrous zirconia precursors were analyzed by PXRD without further treatment following precipitation. Complete uptake of the dopants was confirmed by analysis of the supernatant solution by ICP-MS for Eu and LSC for Cm, with the concentrations being below the detection limit for each method. Thereby, the targeted dopant concentrations in the zirconia solid phases of 30 ppm (Cm^3+^) and 500 ppm or 1 mol% (Eu^3+^) can be confirmed. All precursors were X-ray amorphous. With increasing aqueous synthesis time at the chosen synthesis pH and temperature of 12 and 80 °C, respectively, a phase transformation of the amorphous phase to crystalline zirconia takes place. The diffractograms for selected Eu^3+^- and Cm^3+^-doped samples are compiled in Figure S1. For the Eu^3+^-containing samples, the crystalline fraction of the material could be quantified with known amounts of silicon as internal standard, while the ZrO_2_ phase composition was obtained via Rietveld refinement. Due to the very similar reflection positions of the tetragonal (t) and cubic (c) phases, and the relatively broad Bragg reflections, a distinction between these phases could not be made. The Rietveld refinement was thus performed considering only the monoclinic and tetragonal ZrO_2_ modifications. Results of the refinement have been compiled in Table S1 in the SI. The overall quantity of these higher symmetry polymorphs (t + c) is given in the following text. The quantity of crystalline ZrO_2_ in the Eu^3+^-doped samples and the phase composition of the Eu^3+^- and Cm^3+^-doped samples are shown in Fig. 1. The transformation of the amorphous precursor to the crystalline material increases notably after 10 and 16 days for samples containing 500 ppm (Fig. 1a) and 1 mol% Eu^3+^ (Fig. 1b), respectively. In both cases, the samples undergo almost full crystallization within a few days, reaching > 80% crystallinity after 21 days (500 ppm Eu) and 32 days (1 mol% Eu). For both dopant concentrations, the samples eventually fully crystallize after a hydrothermal synthesis time of > 1 year. Immediately after the initial crystallization, all Eu^3+^- and Cm^3+^-doped samples show a mixture of both the monoclinic and the t + c modifications of zirconia (Fig. 1a–c, red filled circles). With increasing synthesis time, the monoclinic crystal structure (Fig. 1a–c, black filled squares) becomes dominant, accounting for almost 80% of the phase composition, while the t + c phases constitute the remaining 20% of the overall composition for the doped samples.

The crystallite size of all samples was determined using the Debye–Scherrer Eq. (1) and is given in Table 1.

**Table 1:** Crystallite size of doped ZrO_2_ powders derived from the XRD data using Eq. (1).

Dopant (concentration)	Synthesis time (days)	Crystallite size (nm)	
		Monoclinic	Tetragonal^a^
Eu (500 ppm)	16	15 ± 5	11 ± 4
	21	13 ± 3	12 ± 1
	32	14 ± 2	12 ± 2
	63	15 ± 2	11 ± 1
	140	16 ± 3	15 ± 1
	460	17 ± 4	16 ± 1
Eu (1 mol%)	10	13 ± 4	9 ± 2
	16	12 ± 1	12 ± 1
	32	17 ± 2	17 ± 1
	112	18 ± 4	18 ± 1
	399	19 ± 4	18 ± 1
Cm (30 ppm)	16	14 ± 1	13 ± 6
	32	16 ± 1	15 ± 7
	117	18 ± 1	15 ± 3

^a^The cubic modification ($Fm\overline{3}m$) was not used for the refinement due to the peak overlap with the tetragonal modification.

A slight increase of the crystallite size can be seen with increasing hydrothermal treatment time for all dopants and concentrations. The different ZrO_2_ polymorphs and the chosen dopant concentrations, however, do not have a significant effect on the crystallite size. Moreover, the crystallite size seems to be independent on the dopant element considering the similar crystallite sizes for the Eu- and Cm-doped ZrO_2_.

### Transmission electron microscopy

**Figure 2:**
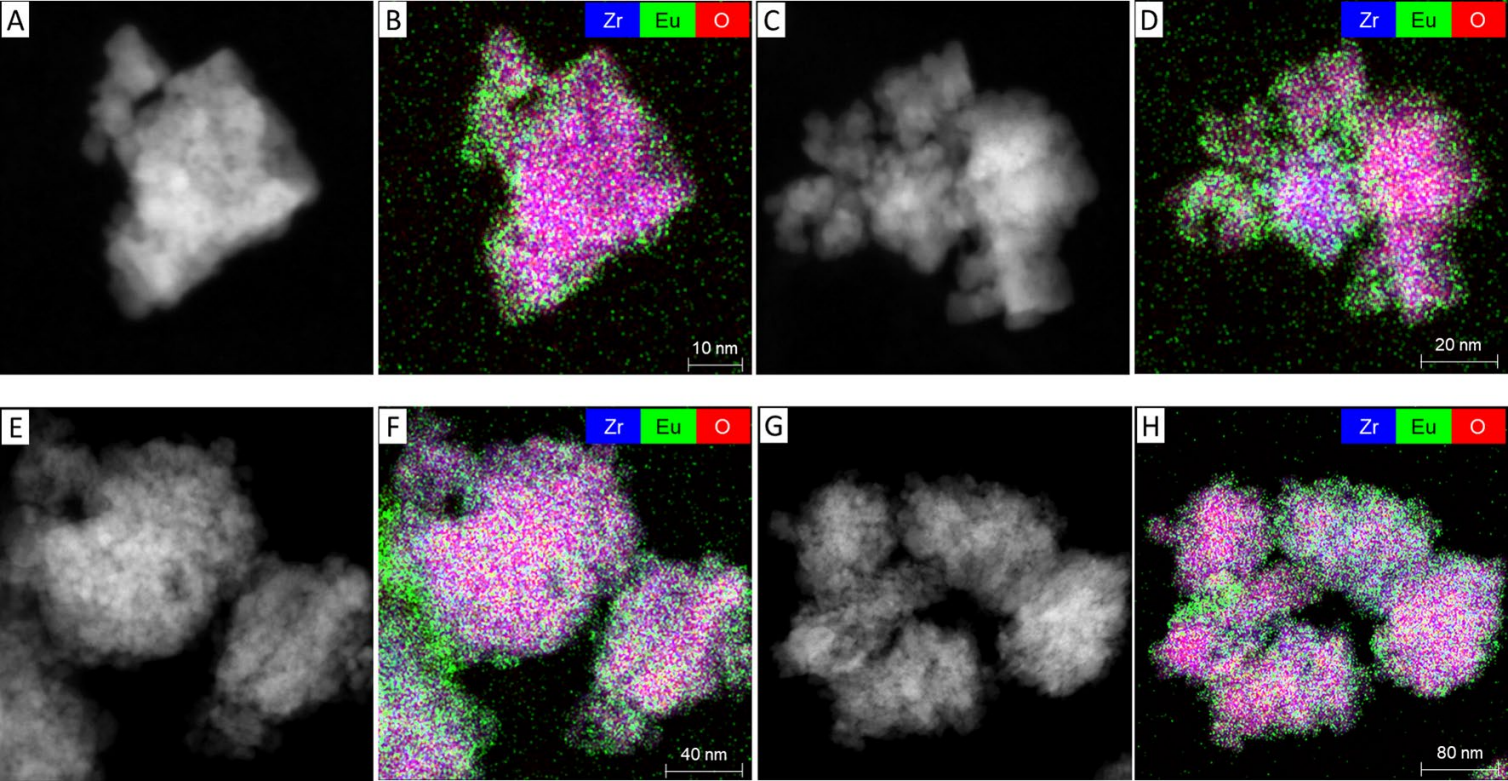
STEM-based analysis for the Eu:ZrO_2_ samples containing 1 mol% dopant after 32 days (**A**–**D**) and 399 days (**E**–**H**) of thermal treatment time. (**A**,**C**,**E**,**G**) are HAADF-STEM images, while (**B**, **D**, **F**, **H**) show the corresponding superimposed EDXS-based element distribution maps for Zr (blue), Eu (green), and O (red).

The morphology and element distribution in the ZrO_2_ samples treated for 32 days and 399 days with 1 mol% Eu were investigated with STEM-based analyses. Looking at the HAADF-STEM images in Fig. 2A,C,E,G, ZrO_2_ seems to form agglomerates which are mainly composed of smaller, rather rounded particles having a size in the nanometer range. With increasing reaction time, the agglomerates tend to grow larger. EDXS-based element mapping (Fig. 2B,D,F,H) of the agglomerates shows a homogeneous distribution of Zr (blue) and O (red) (see also Figs. S2, S3). Regarding Eu (green), there seems to be a preferential accumulation at the periphery of the agglomerates, while a lower amount of europium (compared to the Zr and O signals) is observed in the bulk structure of the material. This is the case for both samples, taken during the crystallization process and after completion of the phase transformation, indicating that accumulation of the Eu dopant at the periphery of the particles takes place during the growth and crystallization and remains there after crystallization has been attained.

### Luminescence spectroscopy

The successful co-precipitation of the dopant cations was confirmed in experiments accounting for surface adsorption or the formation of binary phase mixtures as alternative mechanisms for the removal of Eu or Cm from solution during precipitation. Luminescence spectroscopy enables insights into the structural uptake of a dopant of interest into a solid phase, independent of its crystallinity, and was successfully applied in previous studies regarding nuclear waste forms^28,29^. These experiments are described in detail in the supporting information (section “Luminescence spectroscopy”, Figs. S4, S5) and confirm that the dopants become incorporated into the hydrous zirconia phase during precipitation.

**Figure 3:**
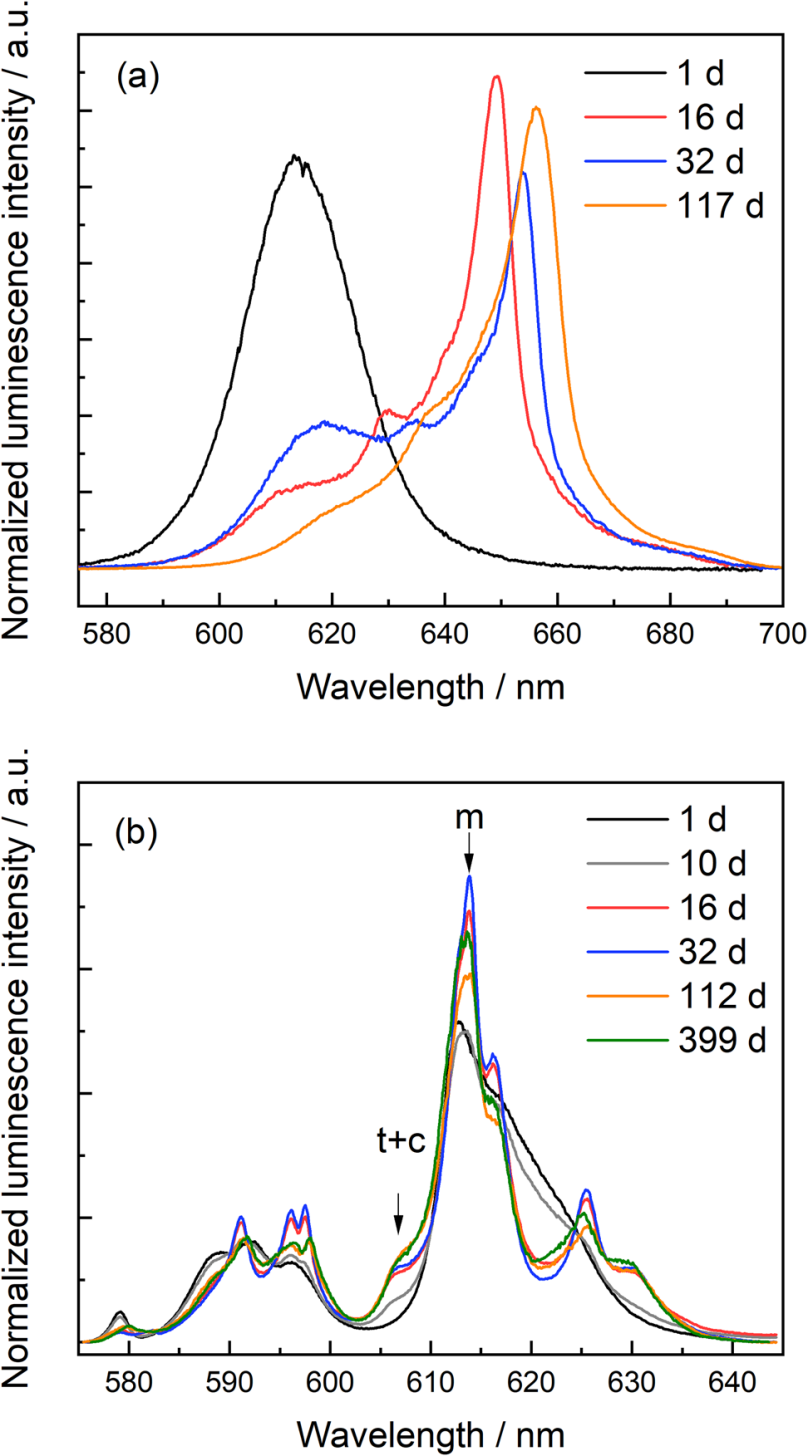
Luminescence emission spectra of the Cm:ZrO_2_ samples containing 30 ppm Cm^3+^ (**a**), and the Eu:ZrO_2_ samples containing 1 mol% Eu^3+^ (**b**). The Cm^3+^ spectra are normalized to the area under the curve, while the Eu^3+^ spectra are normalized to the overall intensity of the ^7^F_1_ band (magnetic dipole transition, 583–603 nm). Unique features for different crystal structures are indicated with arrows (*m* monoclinic, *t* tetragonal, *c* cubic).

To get a first impression of the speciation or speciation changes of Eu^3+^ and Cm^3+^ within the zirconia structure at different aqueous synthesis times, luminescence emission spectra were collected at room temperature following indirect laser excitation in the UV-range (λ_ex_ = 394 nm for Eu^3+^ and λ_ex_ = 396.6 nm for Cm^3+^). The luminescence emission spectra of the ZrO_2_ samples doped with 30 ppm Cm^3+^ (Fig. 3a) and 1 mol% Eu (Fig. 3b) at selected aqueous synthesis times up to 399 days are shown in Fig. 3.

The emission spectra of all synthesized Eu:ZrO_2_ (500 ppm and 1 mol%) samples can be found in the supporting information (Figs. S6, S7). In the beginning of the synthesis where the solid phases are dominated by amorphous material, all Eu-doped samples show similar emission spectra independent of their dopant concentration. The signals of the ^7^F_0-2_ $\leftarrow$ ^5^D_0_ transitions are very broad, consistent with Eu incorporation in an amorphous solid phase. Following spectral decomposition of the recorded composite spectra, using the different luminescence lifetimes of the incorporated Eu species, pure component spectra for two non-equivalent Eu species could be obtained Figure S8. The luminescence lifetimes of 364 ± 22 μs and 1010 ± 125 μs were obtained from the decay curves (Figure S9). These lifetimes can be correlated with the number of H_2_O/OH^−^ entities directly coordinating to Eu, using Eq. (2). The shorter lifetime corresponds to 2.3 water molecules in the Eu coordination sphere, while the longer lifetime indicates an almost complete loss of hydration water, with 0.4 remaining hydration water molecules in the first coordination sphere. With Eu incorporated in a hydrous solid structure, the rather short lifetimes can be better described by O–H vibrational quenching from the hydrous zirconia precursor rather than from coordinating water molecules from the aqueous solvent. As the hydrous zirconia structure can be described by the presence of several non-equivalent cation sites, which differ in their coordination to water of hydration in the amorphous structure, the presence of several non-equivalent Eu species incorporated at these different host cation sites can be understood^30^. Consistent with the Eu-doped samples, two non-equivalent species with different luminescence lifetimes were found for the Cm-doped samples (Figure S5). These lifetimes correspond to 5.1 and 1.6 hydration water entities coordinating to the Cm^3+^ cation, which speaks for an enhanced quenching of the water of hydration in the amorphous solid phase in comparison to Eu^3+^.

With increasing synthesis time and increasing crystallinity of the solid phase, the bands in the Eu^3+^ emission spectra become narrower, and the Stark splitting of the ^7^F_1_ and ^7^F_2_ bands becomes visible (Fig. 3b). The ^7^F_2_ band (605–640 nm) in the recorded emission spectra show a prominent peak at approximately $\lambda$ = 613 nm. This peak is indicative of Eu incorporation into monoclinic ZrO_2_^31–33^. With the onset of the formation of the crystalline zirconia structures, a small peak on the blue side of the ^7^F_2_ $\leftarrow$ ^5^D_0_ transition, at around $\lambda$ = 607 nm becomes visible. This peak has been shown in several studies to arise from Eu^3+^ incorporation in the tetragonal or cubic ZrO_2_ modifications^31,33–35^. Eu^3+^ in the monoclinic environment appears to reach its maximum for the 32 d sample. At longer aqueous synthesis times, a slight decrease of the 613 nm peak intensity can be observed, while the shoulder at 607 nm increases and the bands in the spectra become broader.

Similarly, the Cm spectra (Fig. 3a) show clear narrowing in comparison to the spectrum recorded for Cm^3+^ in the hydrous zirconia precipitate, with increasing aqueous synthesis time, pointing toward an increasingly ordered local environment around the Cm^3+^ cation with increasing crystallinity of the solid phase. The emission peak narrowing is accompanied by an extremely large red-shift of the spectra, to approximately 650 nm. To the best of our knowledge, such a large shift has never been observed before for Cm^3+^ incorporation in a solid matrix. The large shift implies a strong perturbation of the energy levels for Cm incorporated in the crystalline ZrO_2_ environment, which could be caused by an exceptionally short Cm–O bond, and/or a slight covalent character of the bond^36^. Understanding the underlying reason for this extreme perturbation, however, is not the scope of the present study and will be explored in detail in further studies. Although the incorporation of Cm^3+^ in the different structural polymorphs (monoclinic vs. tetragonal or cubic) cannot be deduced from the recorded emission spectra, the strong red-shift of the spectra excludes surface sorption as an uptake mechanism under the applied synthesis conditions^37^. Moreover, the emission peak width of the 32 days sample is narrower than the peak width after 117 days aqueous synthesis time, which is consistent with the Eu^3+^ data discussed above, and points toward the presence of several Cm^3+^ environments in these solid phases.

For a better understanding of the local environment of the trivalent cations in the ZrO_2_ crystal structure, site-selective laser excitation experiments were conducted at 10 K for selected crystalline samples. In these experiments, tunable laser excitation is used to selectively probe non-equivalent species in the solid matrix. For Eu^3+^, the electrons are directly and selectively promoted from the ^7^F_0_ ground state to the emitting ^5^D_0_ excited state. This technique was performed on the 500-ppm-doped samples after 32 d and 460 d and the 1 mol%-doped samples after 32 d and 399 d aqueous synthesis times. For Cm^3+^, laser excitation from the ^8^S′_7/2_ ground state to the ^6^D′_7/2_ excited state, typically denoted with the letter A, was used. The excited A-state of the curium ion undergoes crystal field splitting, giving rise to a maximum of four crystal field states A_1_−A_4_. Selective laser excitation to all these crystal field states was performed for samples synthesized for 32 days and 117 days.

**Figure 4:**
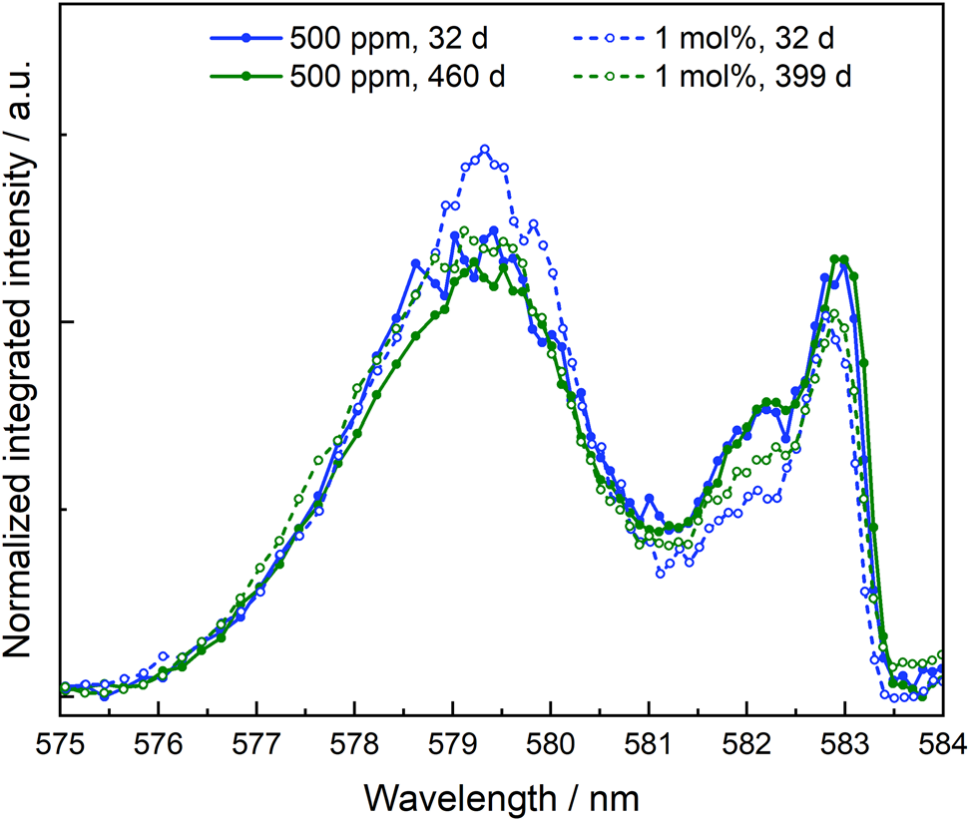
Excitation spectra of Eu:ZrO_2_ with 500 ppm Eu^3+^ (solid lines and filled symbols) and 1 mol% Eu^3+^ (dashed lines, open symbols) after 32 d (blue) and 460 or 399 d (green) aqueous synthesis times.

The excitation spectra of the crystalline samples for both Eu concentrations and synthesis times are shown in Fig. 4.

**Figure 5:**
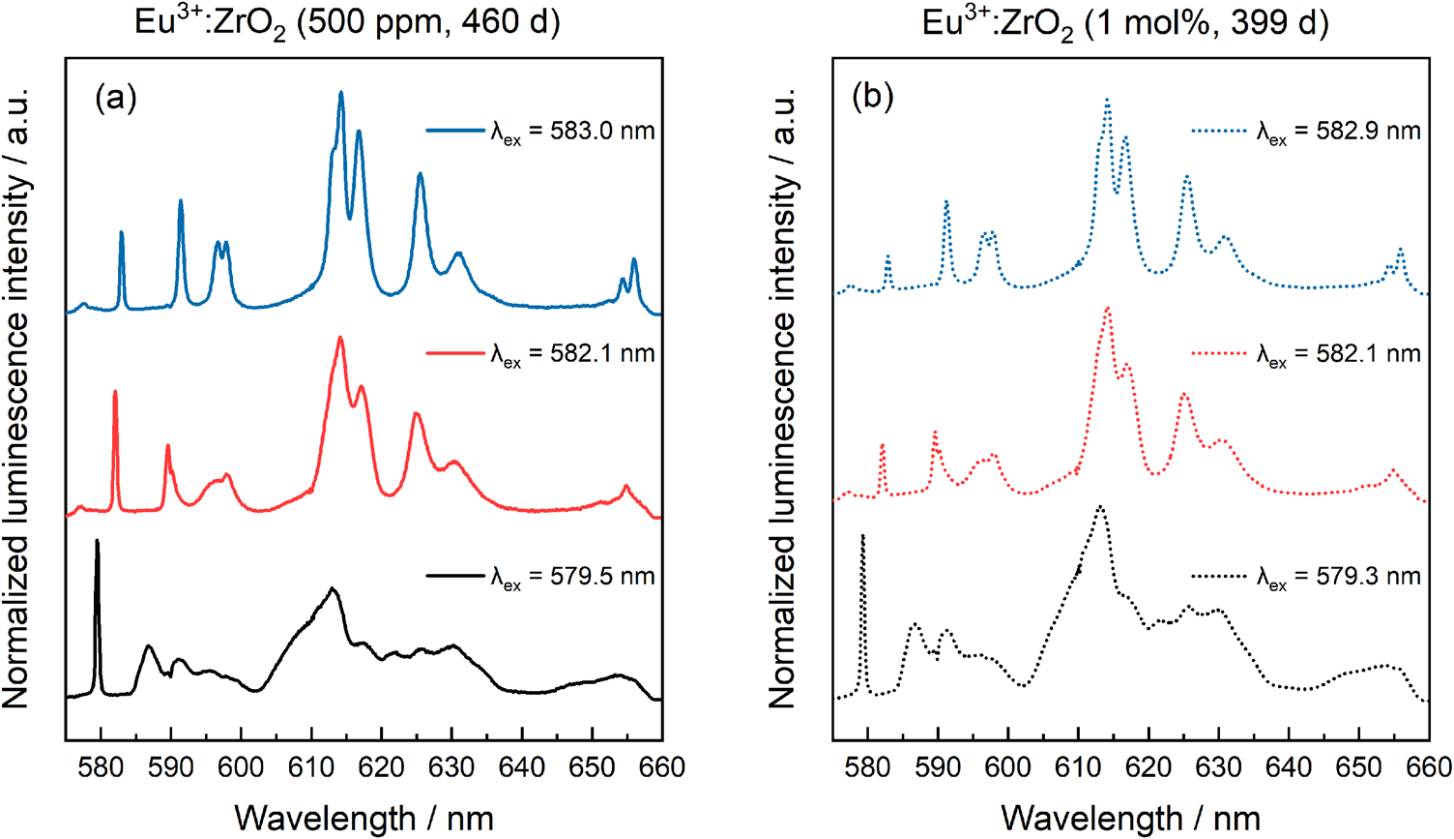
Emission spectra collected at different excitation wavelengths for Eu:ZrO_2_ samples containing 500 ppm Eu^3+^ (**a**, solid lines) and 1 mol% Eu^3+^ (**b**, dashed lines) after 460 and 399 days.

All samples show a broad peak around ~ 579.5 nm and a narrow peak at ~ 582.9 nm with a shoulder at lower wavelengths ~ 582 nm. The excitation spectra after 32 days as well as 460 days are almost identical for the 500 ppm concentration series indicating that once ZrO_2_ has almost completed crystallization, the Eu environment in the solid structure does not change. The 1 mol% concentration sample series shows the same three peak positions, however, with slightly lower contribution of the more red-shifted signals at 582 nm and beyond. Emission spectra at the corresponding excitation peak maxima for both europium concentrations are shown in Fig. 5.

The emission spectrum at 579.5 nm is very broad considering that the host structure is fully crystalline, and the luminescence spectroscopic investigations are conducted in a He-cooled cryostat to reduce thermal broadening of the luminescence signal. When recording a spectrum after a delay time of 8000 μs, a clear emission band belonging to the F_0_–F_2_ transition, at 607 nm, indicative of Eu^3+^ incorporation in a tetragonal/cubic environment, can be seen (Figure S10). This implies, that the broad excitation spectrum is characterized by a short-lived and long-lived species in the tetragonal/cubic crystal structure, which after the delay shows the emission of the longer-lived species only. In both cases, the emission spectrum shows maximum Stark splitting of the emission bands, indicative for Eu incorporation on a lattice site with low symmetry^38^. For the tetragonal and cubic crystal structures, reduced splitting is expected due to symmetry induced degeneracy. This shows that the Eu^3+^ environment in the crystal structure does not correspond to the tetragonal or cubic bulk structure due to lattice distortions caused by the difference in ionic radii of Eu and Zr, which are 1.066 and 0.84 Å for a coordination number of eight, respectively^39^. In addition, one oxygen vacancy is generated in the ZrO_2_ structure for every two incorporated europium ions, to preserve charge neutrality in the crystal structure. The presence of these vacancies are likely to decrease the local order, and consequently the local symmetry of the solid matrix.

The two peaks at higher wavelengths (582.1 and 582.9 nm) are associated with Eu incorporated into the monoclinic crystal structure, based on the emission spectra recorded at these excitation wavelengths, showing the characteristic ^7^F_2_ peak at 613 nm. Since the monoclinic crystal structure only has a single cation site that can be occupied by a dopant ion, the two detected monoclinic environments must have some fundamental differences. Both excitation peaks at 582.1 and 582.9 nm differ only minimally in their emission spectra, mainly in the relative intensities of the ^7^F_1_ and ^7^F_2_ bands. Thereby, the two Eu^3+^ environments are very similar, and may differ only with respect to their coordination to the surrounding oxygen anions and created oxygen vacancies, as previously found for Eu^3+^ incorporated in cubic ZrO_2_^34^. More specifically, a coordination number of seven in the pristine monoclinic ZrO_2_ environment would arise when the oxygen vacancy is not directly coordinated to the Eu^3+^ cation, while a reduction of the coordination number to six would occur when a vacancy is directly coordinated to the incorporated Eu^3+^ cation. The vacancy is known to be slightly larger than the oxygen anion, thereby increasing the unit cell volume and, subsequently increasing the M–O distance (M = Zr^4+^ or Eu^3+^)^40^. Thereby, we assign the Eu^3+^ environment at 582.1 nm to Eu^3+^ incorporation in monoclinic ZrO_2_ with direct coordination to an oxygen vacancy, while the species at 582.9 nm is Eu^3+^ in a pristine m-ZrO_2_ environment.

Based on the area under the curve in the excitation spectra, the dopant shows preferential incorporation into the tetragonal/cubic structure although the monoclinic crystal structure is dominant in the samples based on XRD results after the beginning of crystallization (Fig. 1). More specifically, approximately 70 ± 5% of the Eu^3+^ signal can be attributed to the c + t phase, and the remaining 30 ± 5% to Eu^3+^ in m-ZrO_2_. This observation cannot be made by using UV excitation (Fig. 3b). At room temperature (UV excited spectra), all present Eu^3+^ species are simultaneously excited with the laser light. Radiationless relaxation from a higher-lying excited state to the emitting excited state occurs, and energy transfer processes, for example from the cubic/tetragonal phase to the monoclinic phase can occur, which is likely the reason for the overrepresentation of the monoclinic environment using this method. Alternatively, this could be explained by a lower transition probability for Eu in the cubic/tetragonal structure and a higher, and therefore overrepresented, probability of the transition for the monoclinic phase.

**Figure 6:**
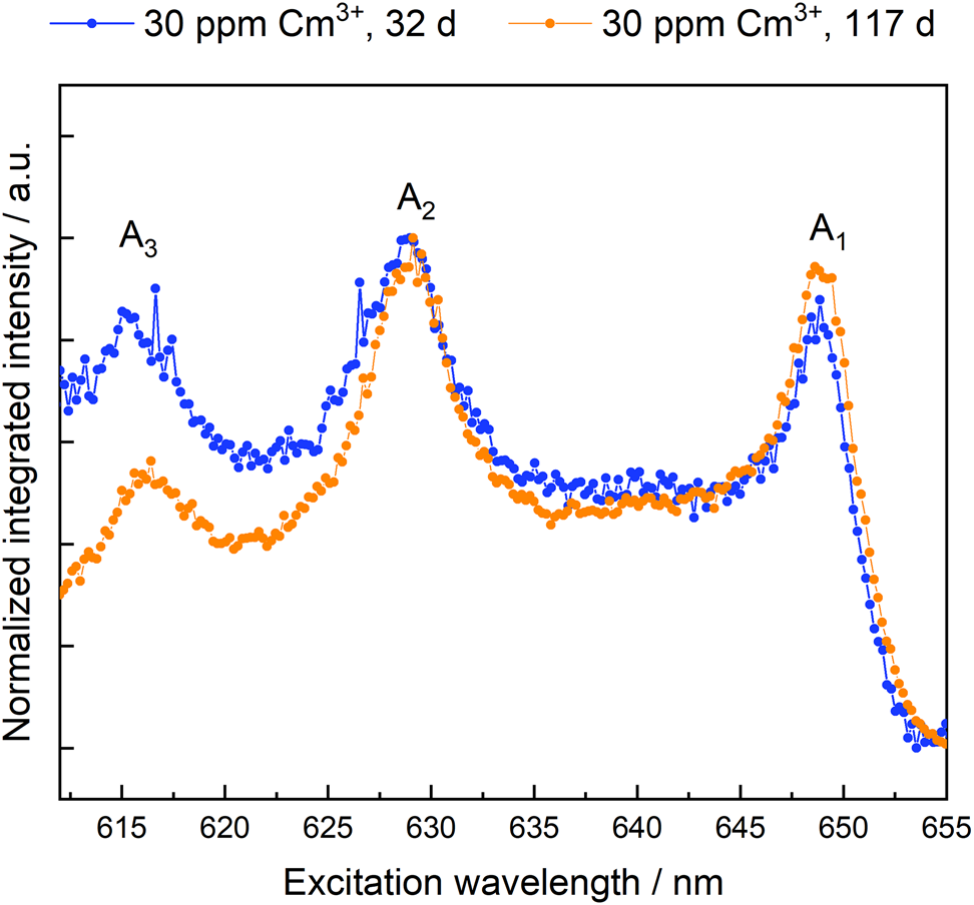
Excitation spectra of the 30-ppm Cm:ZrO_2_ samples after 32 days (blue) and 117 days (orange) aqueous synthesis times.

The excitation spectra of the Cm:ZrO_2_ samples for 32 days and 117 days hydrothermal treatment times are shown in Fig. 6.

**Figure 7:**
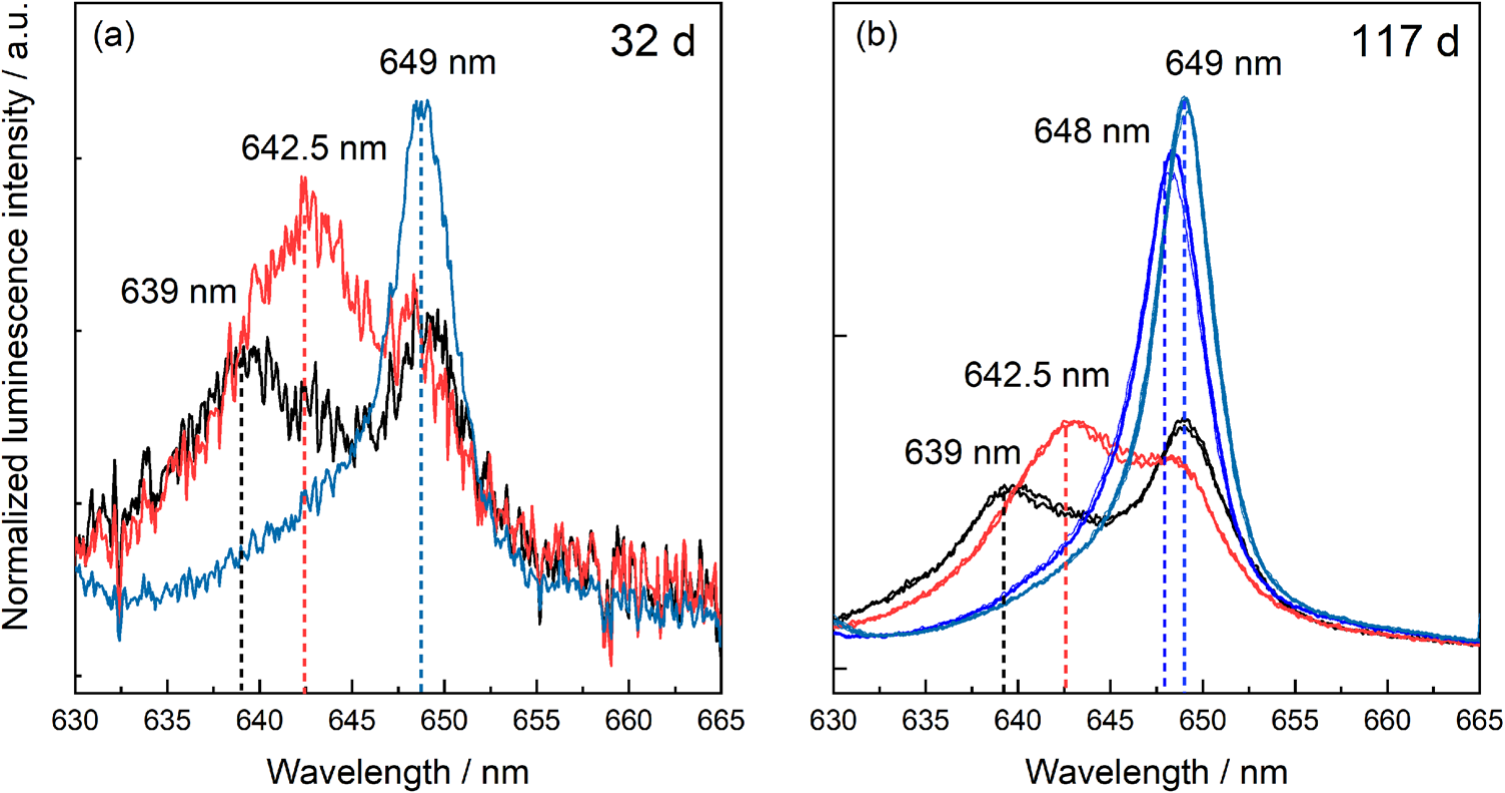
Emission spectra of ZrO_2_ samples doped with 30 ppm Cm^3+^ after 32 days (**a**) and 117 days (**b**) collected at different excitation wavelengths between 623 and 630 nm (excitation to A_2_ level).

The excitation spectra are characterized by three peaks. Peaks A_3_ and A_2_ are crystal field states of the ^6^D′_7/2_ term with higher energy, usually referred to as hot-band transitions, while the peak labeled A_1_ is the crystal field level with the lowest energy, from which luminescence emission back to the ground state occurs. Contrary to the sample crystallinity, the excitation peak of the 32 days sample is slightly narrower than for the sample subjected to a longer aqueous synthesis time. The luminescence emission spectra collected via excitation directly to the emitting crystal field state (A_1_) show extreme line-narrowing effects (see Figure S11), which can be explained by a continuum of very similar environments arising from the lack of long-range order in the solid structure. Direct excitation to the hot-band transition (A_2_), avoiding the line narrowing effect, reveals multiple Cm^3+^ environments for the 32 days and 117 days samples, Fig. 7.

The overall luminescence intensity of the 32 days sample is poor in comparison to the 117 days sample. However, in both cases two broad peaks with emission peak positions at approximately 639 nm and 642.5 nm can be identified. These emission peak positions are consistent with Cm^3+^ incorporation in a t + c ZrO_2_ environment^41^. The narrower peak at 649 nm (32 days sample) or 648 nm and 649 nm (117 days sample) are thereby assigned to Cm^3+^ in a monoclinic ZrO_2_ structure, in accordance with the Eu^3+^ luminescence data. The fact that the peak positions of Cm^3+^ incorporation in m-ZrO_2_ are almost identical, points toward very similar coordination spheres which in turn is consistent with Cm^3+^ coordination to seven oxygen anions or six anions and one vacancy, respectively.

## Discussion

### Crystallization and phase stabilization

The amorphous zirconia structure containing Eu starts crystallizing after approximately 10 days at a constant synthesis pH and temperature of 12 and 80 °C, respectively (Fig. 1). Other hydrothermal zirconia studies showed that at elevated temperatures of $\ge$ 95 °C, this initial transformation occurs much faster, sometimes already after a few hours^20,22,42^. This indicates a temperature dependence of the crystallization rate, which to some extent can be attributed to the slightly lower solubility of the hydrous zirconia precursor at lower temperatures^43^. Additionally, the dissolution/precipitation reaction is strongly pH-dependent. The reported solubility of the hydrous zirconia precursor is several orders of magnitude lower in the near-neutral pH range than for very acidic or alkaline systems^43,44^. According to the studies of Clearfield and Denkewicz et al., the formation of the different zirconia modifications depends on the starting pH^43,45^. The thermodynamically stable monoclinic zirconia modification is formed over the entire pH range, while the tetragonal and cubic phases are only observed in alkaline systems. Over time, the tetragonal structure undergoes a transformation into the thermodynamically stable monoclinic modification^43^. Other studies show that the dopant concentration influences the kinetics of the tetragonal to monoclinic transformation during hydrothermal aging^46^. Lower dopant concentrations tend to increase the transformation rate from the meta-stable tetragonal to the monoclinic phase. The dopant size also plays a crucial role in the kinetics of the transformation. Dopants with a larger size mismatch to the zirconium host were shown to have a much slower conversion rate than dopants having a similar ionic radius. In all cases, the tetragonal to monoclinic transformation reached a maximum conversion at which no further material underwent phase transformation. This value was reported to be between 80 and 85% for the material that underwent phase transformation from tetragonal to monoclinic. Higher dopant concentrations reduce that value due to lattice stabilization caused by the dopant^46,47^.

In the current study, no phase transformation of a meta-stable tetragonal phase was observed over the course of > 1 year. In contrast, the amount of the t + c phases increases slightly but constantly over time for the samples with 500 ppm Eu^3+^ or 30 ppm Cm^3+^ (Fig. 1). This is likely related to the small size of the crystallites in all samples (Table 1). It has been shown that the tetragonal to monoclinic phase transformation is suppressed below a certain particle size threshold, thereby stabilizing the tetragonal phase. An in-depth study by Garvie^18^ investigated the stability of tetragonal crystallites formed after precipitation from aqueous solution. Shukla and Seal^19^ summarized Garvie’s study amongst others to show the impact of various effects (i.e. surface energy, interfacial energy, strain energy, anions and more) on the phase stability. They concluded a critical crystallite size of 10–33 nm before the tetragonal phase would become meta-stable and transform into the monoclinic phase^14,18,19^. The maximum tetragonal crystallite size in this study is 18 nm (Eu:ZrO_2_, 1 mol%, 112 days), i.e. well within the reported range resulting in a stable tetragonal or t + c phase in the samples. Prolonged synthesis times of up to 460 days do not affect the crystallite size, which would explain the absence of a tetragonal to monoclinic phase transformation. The stability of the tetragonal phase can be attributed to the role of interfacial energy caused by the agglomeration and modification of the surface energy of the zirconia particles^48^, with the tetragonal phase remaining stable under these hydrothermal conditions. Besides mere size effects, subvalent dopants are known to stabilize the tetragonal and cubic modifications. This stabilization mechanism is attributed to the reduction of the coordination number, especially for the small Zr^4+^ host cation, via the introduction of oxygen vacancies following trivalent cation incorporation, to preserve charge neutrality in the crystal structure. Especially in the sample with 1 mol% Eu^3+^, the occurrence of the t + c modifications in the ZrO_2_ samples can be reasonably assumed to arise from both crystallite size effects and the above-discussed stabilization by the subvalent dopant^49^.

### Dopant incorporation species

Existing studies have reported phase segregation of dopants from stabilized zirconia structures during hydrothermal conditions. For example, Zhang et al.^46^ reported immediate phase segregation of oversized (Nd, La) or undersized dopants (Al) from the ZrO_2_ matrix for dopant concentrations of 0.6–0.8 mol%^48^. According to the combined microscopic and spectroscopic investigations, these findings were not confirmed in the present study, using oversized trivalent dopants in concentrations ranging from 30 ppm to 1 mol%. Phase segregation would lead to either an accumulation of the trivalent cations on the ZrO_2_ surface as adsorption complexes, or the precipitation of the dopant hydroxides or hydroxycarbonates from oversaturated solutions. Both adsorption and precipitation processes could be excluded based on the luminescence spectroscopic investigations described in the supporting information (section “Luminescence spectroscopy”, Figs. S4, S5). For the highest Eu^3+^ dopant concentration of 1 mol%, our STEM-EDXS data shows accumulation of the trivalent cation close to or at the surface of the ZrO_2_ particle agglomerates (Fig. 2), which we will refer to as superficial dopant species in the following text. A relatively lower Eu^3+^ concentration can be detected within the agglomorates, i.e. in the bulk (Figs. S2, S3). The amount of superficial vs. bulk species does not increase over aqueous synthesis time, but the distribution of the dopant within the zirconia matrix seems to remain the same once crystallization of ZrO_2_ has started. By examining the luminescence data for the Eu^3+^- and Cm^3+^-doped ZrO_2_ samples with increasing aqueous synthesis time, a complex but unchanged speciation of the *f*-element cations in the solid structure can be deduced. From the Eu^3+^ luminescence data, a clear preference for incorporation within the t + c modifications over the monoclinic one can be seen, based on the area under the measured excitation spectra shown in Fig. 4, Eu^3+^ incorporation in the monoclinic phase amounts to approximately 30 ± 5% of the Eu^3+^ speciation in the crystalline ZrO_2_ solid material, as deduced from the combined integrals of the peaks at 582.1 and 582.9 nm relative to the overall integral of the measured spectra. The amount of Eu^3+^ associated with the higher symmetry polymorphs, characterized by the broad signal at approximately 579.5 nm in Fig. 4, is 70 ± 5%. This amount is further divided between two Eu^3+^ environments which differ in their luminescence decay times. The presence of a short-lived Eu^3+^ species, which is not adsorbed on the ZrO_2_ surface, could arise from e.g. a Eu^3+^ ion partially coordinating to water in the surrounding solution or from Eu^3+^ coordination to surface-terminated hydroxyl groups at the very superficial sites in the ZrO_2_ particles. The Eu^3+^ species with a significantly longer luminescence lifetime is indicative of complete isolation of Eu^3+^ from the surrounding aqueous solution, which can be attributed to Eu^3+^ incorporation into the c + t-ZrO_2_ bulk material. Based on the combined microscopic and spectroscopic results, we assign the short-lived Eu^3+^ species to the visible Eu^3+^ accumulation at the periphery of the agglomerates. Whether the accumulation can be attributed to an overall smaller size of the particles, thereby increasing the surface-to-bulk ratio, can unfortunately not be deduced from our TEM-based analysis due to the agglomerate formation preventing the unambiguous identification of individual ZrO_2_ particles.

Finally, Eu^3+^ and Cm^3+^ incorporation within the monoclinic crystal structure is characterized by the presence of at least two non-equivalent species with clearly narrower luminescence spectra than observed for incorporation in t + c ZrO_2_. This is rather surprising, as the monoclinic crystal structure only has one crystallographically unique Zr^4+^ cation site and, consequently, only one Eu^3+^ and Cm^3+^ environment would be expected. The Eu^3+^ emission spectra collected via selective excitation at the peak maxima of the two non-equivalent environments in the monoclinic modification, however, are very similar (Fig. 5b), pointing toward very subtle differences between these species. One potential explanation for the presence of two non-equivalent species with very similar emission spectra is the presence of oxygen vacancies in nearest-neighbor or next-nearest-neighbor positions with respect to the incorporated dopant. The position of the generated oxygen vacancies with respect to Eu^3+^ is rather well-described for Eu^3+^ incorporation in the cubic ZrO_2_ polymorph, but has hitherto not been described for the monoclinic structure^34,50,51^. Although a definite assignment of the different trivalent environments in the monoclinic ZrO_2_ structure would require additional studies, the formed species were observed to be stable and do not change much during extended periods of aqueous synthesis. We can thereby conclude, that the incorporation of trivalent *f*-elements in the zirconia polymorphs formed after crystallization show a high stability without any signs of dopant segregation from the crystal structure.

## Conclusion

This study investigated the role of ZrO_2_, a corrosion product forming on the nuclear fuel rod cladding surface, to act as engineered barrier material via structural incorporation of potentially mobilized radionuclides in a DGR. Experimental conditions such as elevated temperature and solution chemistry were chosen to mimic conditions that may arise in a generic DGR as a result of radioactive decay and the corrosion of the canister and cementitious backfill and sealing materials. Our experiments revealed a successful co-precipitation of Eu^3+^ as well as Cm^3+^ with zirconia, forming an amorphous hydrous precursor containing multiple incorporation sites of the dopant elements at the initial stage. Upon progressing synthesis duration, the material underwent crystallization yielding a mixture of mainly the thermodynamically stable monoclinic structure and a smaller fraction of the tetragonal and cubic polymorphs. The occurrence of the tetragonal and cubic phases arises from the structural stabilization due to the introduction of oxygen vacancies reducing the coordination number of the Zr lattice as well as the small crystallite size (< 20 nm). The various phases were stable and did not undergo a phase transformation, most likely due to agglomeration of crystallites, thus modifying the surface energy. Immediately after crystallization, the trivalent dopants could be located in the different crystal structure polymorphs. Although the phase distribution showed that most of the zirconia crystallized in the monoclinic phase, for Eu, a preferential incorporation into the tetragonal and cubic structure was observed. In the case of the Cm incorporation, this preference for the high-temperature polymorphs was not evident. Over the entire synthesis duration of up to 460 days, the dopant remained structurally incorporated in the crystal structure and did not undergo phase segregation. TEM-based analysis revealed a clear accumulation of the dopant at the particle peripheries resulting in an additional dopant environment. This environment in characterized by a clearly shorter luminescence lifetime in comparison to incorporated species, either as a result of direct Eu^3+^ coordination to water in the surrounding solution or from Eu^3+^ coordination to surface-terminated hydroxyl groups at the very surface of the ZrO_2_ particles. Independent of the crystal structure, the dopant resides in a superficial and bulk environment. Complementary XRD, STEM and luminescence spectroscopy measurements revealed trivalent cations to be structurally embedded into ZrO_2_ during slightly elevated temperature hydrothermal experiments. These results indicate that ZrO_2_, present as cladding corrosion material of spent fuel rods in a DGR, would additionally be suitable to act as engineered barrier material for immobilization of mobilized trivalent cations by structural uptake of these mobilized species, in case of water intrusion into a DGR. Future studies will focus on the role of pH towards the immobilization of trivalent actinides within the different zirconia polymorphs. In addition, spectroscopic investigations of Eu^3+^- and Cm^3+^-doped monoclinic ZrO_2_ obtained via high-temperature calcination routes are planned to elucidate the origin of multiple dopant environments in the m-ZrO_2_ crystal structure and the unusually large spectral shifts acquired for the Cm^3+^-doped material.

## Materials and methods

### Materials

#### Aqueous synthesis of M^3+^ (M = Eu, Cm)-doped ZrO_2_

##### Precipitation

M^3+^-doped hydrous zirconia samples were prepared by dissolving ZrOCl_2_·8 H_2_O (Sigma-Aldrich, > 99.99%) in 0.01 M HCl using a 1:2 ratio mass (g)/volume (mL), resulting in a Zr^4+^ concentration of 1.55 M. The trivalent cation was added to the Zr solution from a stock solution of 0.1 M EuCl_3_·6 H_2_O in 1 mM HCl or 0.1 mM ^248^Cm^3+^ in 0.01 M HClO_4_ to obtain the desired dopant concentrations of 500 ppm, 1 mol% (Eu), and 30 ppm (Cm) in the hydrous zirconium precursor. The concentration of the dopants refers to the number of M^3+^ per total M^3+^ + Zr^4+^ cations in doped ZrO_2_, assuming quantitative incorporation of the cations from solution into the formed co-precipitate. Subsequently, this mixed solution was added dropwise under constant stirring to a 0.5 M NaCl solution in a ratio of approximately 1:20 for Eu^3+^ doped and 1:28 for Cm^3+^ doped samples (M^3+^/M^4+^ solution: NaCl). The pH was readjusted to its initial value of 12 with 2 M NaOH. The suspension was left for 24 h at room temperature before the solid phase was recovered via centrifugation of the suspension for 30 min at 4000 rpm. An aliquot of the supernatant was taken for Inductively-Coupled Plasma Mass Spectrometry (ICP-MS for Eu) or Liquid Scintillation Counting (LSC for Cm) analysis to ensure complete uptake of the dopant. In all cases, the dopant concentration in the supernatant was below the detection limit, thereby confirming the quantitative uptake of the dopants by the formed solid phase. The rest of the liquid phase was discarded, and the wet solid was washed twice with MilliQ water (18.2 MOhm). The washing step included addition of 40 mL of MilliQ water into the Greiner tubes, full resuspension of the solid phase, and immediate centrifugation of the suspension, subsequently discarding the washing solution. After the second washing step, the wet solid was dried for 24 h at 80 °C. The dry solid was ground in an agate mortar into a fine powder.

To probe whether the M^3+^ dopant was (1) adsorbed onto the Zr-containing precipitate or (2) formed a separate phase, such as a hydroxide or hydroxy carbonate rather than a co-precipitate, additional samples were prepared. Adsorption of Eu^3+^ on the amorphous precipitate was investigated at pH 12. For this purpose, non-doped hydrous zirconia was precipitated in the absence of Eu^3+^, as described above. 60 mg of the precipitate were thereafter suspended in 40 mL of 0.5 M NaCl at pH 5. 25 µL of a 0.01 M EuCl_3_ $\cdot$ 6 H_2_O stock solution were added. The pH 12 was adjusted gradually over the period of two days by manual addition of small aliquots of NaOH under continuous stirring to avoid large pH-fluctuations and, thereby, to minimize the risk of immediate precipitation of the europium. The samples were then equilibrated at room temperature for 24 h. After the experiment was finished, the suspension was centrifuged for 30 min and the wet solid was dried at 80 °C for 24 h. The pure Eu precipitate was synthesized by dropwise addition of the acidic 0.1 M Eu^3+^ stock solution to a 0.5 M NaCl solution at pH 12. The suspension was centrifuged for 30 min and afterwards washed twice with MilliQ water. The white precipitate was dried at 80 °C for 24 h.

#### Aqueous treatment of M^3+^-containing hydrous zirconia precipitates

For the Eu-doped experiments, 120 mg of the doped zirconia powder was submerged in 40 mL 0.5 M NaCl and the pH was adjusted to 12 with 2 M NaOH. This resulted in a 1:3 ratio volume (mL)/mass (mg). The suspension was transferred into a 50 mL plastic Greiner tube and stored in an oven at 80 °C for the desired amount of time. After the thermal treatment, the sample was taken out of the oven and cooled down to room temperature. The sample was then centrifuged for 30 min and washed with 20 mL MilliQ water twice, following the procedure discussed above. The solid was dried at 80 °C for 24 h and then ground in an agate mortar into a fine powder for further analysis.

For the Cm-containing samples, 100 mg of the powder was submerged in 13.3 mL 0.5 M NaCl with pH values of 5 and 12, resulting in a 1:7.7 ratio volume (mL)/mass (mg). The hydrous zirconia sample at pH 5 was solely used for comparison to adsorption investigations, to elucidate the role of surface adsorption in the removal of *f*-elements from solution in the zirconia precipitation step. Time-dependent hydrothermal studies were only conducted for the samples prepared at pH 12. Afterwards, the samples underwent the same treatment as the Eu-doped samples. All Cm-involving tasks were performed insight a glovebox with negative pressure in N_2_ atmosphere and carbonate-free solutions.

### Methods

#### Powder X-ray diffraction

To follow the crystallization of the ZrO_2_ solid phase as a function of the hydrothermal synthesis time and to identify the structural modifications (monoclinic, tetragonal, cubic) of the crystallized ZrO_2_, Powder X-ray diffraction (PXRD) analyses were performed on a Rigaku MiniFlex 600 using a Cu K_α_ X-ray source (40 kV/15 mA operation for X-ray generation). Samples were finely ground in an agate mortar. The samples containing inactive Eu^3+^ were measured on a silicon wafer in the 2θ range between 5° and 90° with a step size of 0.02° 2Θ/min while spinning the Si wafer. The radioactive Cm-containing samples were prepared inside a glovebox under N_2_ atmosphere using an air-tight sample holder consisting of a Kapton-domed silicon wafer. The Cm-containing samples were measured in a range between 5° and 60° 2Θ with a step size of 0.02° 2$\Theta$/min without spinning to avoid possible contamination of the sample holder. The crystallinity was only determined for the inactive samples by the addition of 20 wt% monocrystalline silicon as an internal standard to each sample, followed by thorough grinding of the sample mixture into a homogenous fine powder. For the Rietveld refinement, references of the monoclinic (space group:$P{2}_{1}/c$) and tetragonal ($P{4}_{2}/nmc$) zirconia modification as well as silicon ($Fd\overline{3}m$) were used in the PDXL2 software (Rigaku Corporation). More specifically, the database cards 04-004-4339 (m-ZrO_2_), 04-005-4479 (t-ZrO_2_), and 04-014-8844 (Si) were taken for the refinement. The cubic zirconia modification ($Fm\overline{3}m$) was not used for the refinement due to the peak width overlapping with the tetragonal modification. However, as we cannot exclude its presence in the synthesized solid phases, the quantity of the refined tetragonal phase is referred to as c + t-ZrO_2_ throughout the text. The background, peak shape, and preferred orientation effects were corrected or accounted for using B-spline, Split pseudo-Voigt, and March–Dollase models in the refinement.

The crystallite size was determined using the Debye–Scherrer Eq. (1):

1 $$L=\frac{K\cdot \lambda }{\Delta \left(2\Theta \right)\cdot cos\Theta }$$

Here, L represents the crystallite size perpendicular to the lattice plane of the Bragg reflection in *nm*. K is a shape factor, which is dependent of the shape of the crystals and the absolute value can only be determined empirically. In the current work, a shape factor of $K=0.9$ was used, in agreement with previous studies^22,42^. The diffractograms were measured using a Cu X-ray source (${\lambda }_{C{u}_{\alpha }}=0.154nm$). $\Delta \left(2\Theta \right)$ is the full width at half maximum (FWHM) of the diffraction maximum with Θ being the Bragg angle.

#### Transmission electron microscopy

To examine the morphology of the ZrO_2_ solid phases following aqueous synthesis and the distribution of the dopants within the solid ZrO_2_ structure, high-angle annular dark-field scanning transmission electron microscopy (HAADF-STEM) imaging combined with spectrum imaging analysis based on energy-dispersive X-ray spectroscopy (EDXS) were performed at an accelerating voltage of 200 kV with a Talos F200X microscope equipped with an X-FEG electron source and a Super-X EDX detector system (FEI). Prior to STEM analysis, the specimen mounted in a high-visibility low-background holder was placed for 2 s into a Model 1020 Plasma Cleaner (Fischione) to remove possible contaminations. The TEM specimens were prepared by creating a very diluted suspension of the sample in MilliQ water and pipetting it onto a carbon-coated TEM grid and letting the water evaporate under ambient atmosphere.

#### Luminescence spectroscopy

The Eu^3+^ and Cm^3+^ environments in the solid phases were investigated via luminescence spectroscopy. Two different spectroscopic approaches were used as explained below.

All synthesized samples were subjected to luminescence spectroscopic investigations at room temperature, using a non-selective excitation wavelength of 394 nm or 396.6 nm, capable of exciting all non-equivalent Eu^3+^ species or Cm^3+^ species, respectively. These non-selective luminescence spectroscopic experiments were performed using a pulsed dye-laser (NarrowScan, Radiant Dyes) with a 1:1 dye mixture of Exalite 389 and Exalite 398, coupled to a Nd:YAG (Continuum, Surelite) pump laser. The Eu^3+^ luminescence emission spectra were recorded in a range between 575 and 645 nm, 1 µs after the exciting laser pulse, while a spectral range between 560 and 700 nm was used for Cm^3+^, 1 µs after the exciting laser pulse. The spectra were recorded using the Andor SOLIS software. The laser pulse energy was measured to be between 2 and 3 mJ. Luminescence emission spectra were detected using an optical multichannel analyzer (Shamrock 303i) with 300 or 600 lines/mm grating and an iCCD camera (iStar, Andor). Delayed spectra for the identification of the Eu^3+^ or Cm^3+^ species with different lifetimes were recorded by delaying the signal detection after the laser pulse up to 15 ms (Eu) or 800 μs (Cm). Luminescence lifetime measurements were performed by collecting multiple spectra and stepwise increasing the time delay of the signal detection and the laser pulse between 20 and 500 µs.

To determine the number of water molecules in the first ligand sphere the equations of Horrocks et al.^52^ (2) and Kimura et al.^53^ (3) can be used:

2 $$q\left(E{u}^{3+}\right)=1.07\cdot \frac{1}{\tau \left[ms\right]}-0.62$$

3 $$q\left(C{m}^{3+}\right)=0.65\cdot \frac{1}{\tau \left[ms\right]}-0.88$$

Based on the results of the non-selective luminescence spectroscopic investigations, the chosen samples were subjected to additional spectroscopic experiments with selective, tunable laser excitations in a He-cooled cryostat < 10 K (Janis and Sumitomo, SHI cryogenics group). The samples were placed in a copper sample holder covered by quartz glass. Therefore, a Nd:YAG pump laser (Continuum SureLite II) was coupled to a dye system (NarrowScan, Radiant Dyes) containing Rhodamine 6G/Rhodamine B (560–585 nm), DCM (600–660 nm), and Sulforhodamine (585–605 nm) dye. An optical multichannel analyzer (Shamrock 303i) with gratings of 150, 600, and 1200 lines/mm and an iCCD camera (iStar, Andor) were used. The laser energy was measured by the help of an energy meter (Newport 1918-R) which gave the mean energy while collecting one spectrum which was later used for normalizing the resulting excitation spectrum. The exact wavelength was measured with a wavelength meter (High Finesse WS-5). All spectra were recorded via the Andor SOLIS software.

## Supplementary Information

Supplementary Information.

## Data Availability

The datasets used and/or analyzed during the current study available from the corresponding authors on reasonable request.
