# Supplementary material for: Investigations towards incorporation of Eu3+ and Cm3+ during ZrO2 crystallization in aqueous solution
Source: Sci Rep. 2023 Jul 28;13:12276. doi: 10.1038/s41598-023-39143-0 (PMC10382555; doi:10.1038/s41598-023-39143-0)
Supplement: Supplementary file 1 — Supplementary Information. [file 41598_2023_39143_MOESM1_ESM.pdf]

Supporting Information

Investigations towards incorporation of Eu^{3+} and Cm^{3+} during ZrO_2 crystallization in aqueous solution

Lucas Opitz[1,2], René Hübner[3], Salim Shams Aldin Azzam[1], Sara E. Gilson[1], Sarah C. Finkeldei[2,4,5*], Nina Huittinen[1,6*]

[1]Institute of Resource Ecology, Helmholtz-Zentrum Dresden - Rossendorf, 01328 Dresden, Germany

[2]Department of Chemistry, University of California, Irvine, Irvine, CA 92697, USA

[3]Institute of Ion Beam Physics and Materials Research, Helmholtz-Zentrum Dresden-Rossendorf, 01328 Dresden, Germany

[4]Department of Materials Science and Engineering, University of California, Irvine, Irvine, CA 92697, USA

[5]Department of Chemical and Biomolecular Engineering, University of California, Irvine, Irvine, CA 92697, USA

[6]Institute of Chemistry and Biochemistry, Freie Universität Berlin, 14195 Berlin, Germany

*corresponding author

PXRD

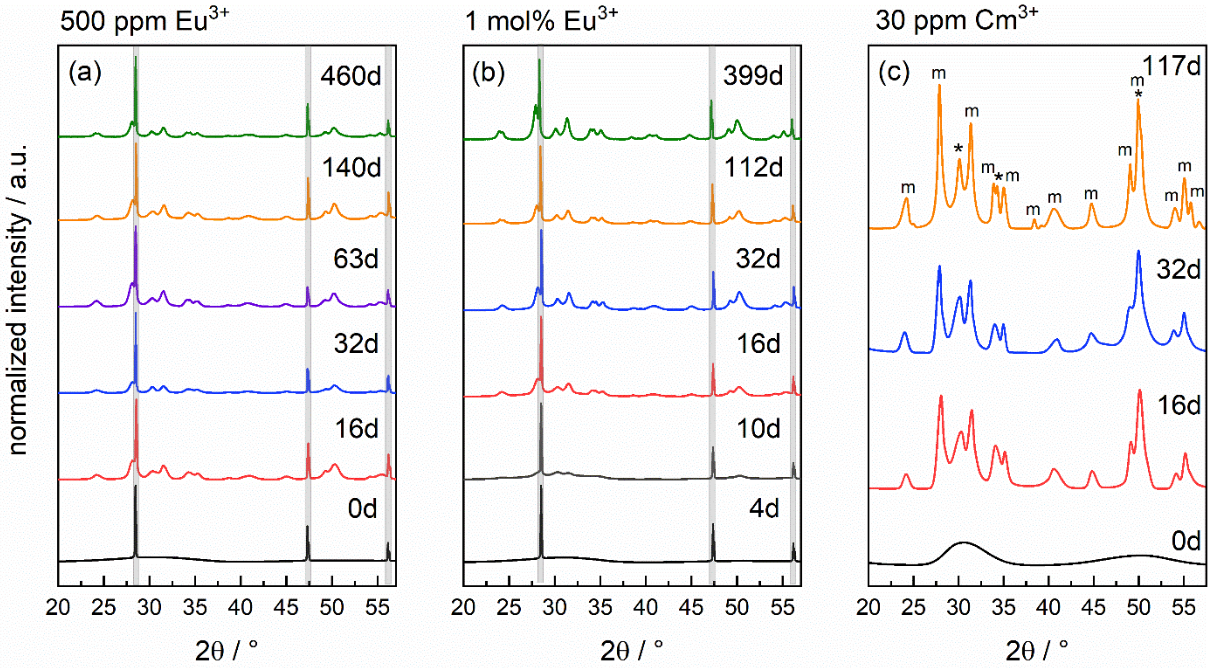

Figure S1: X-ray diffractograms of selected ZrO_2 samples containing (a) 500 ppm Eu^{3+}, (b) 1 mol% Eu^{3+}, and (c) 30 ppm Cm^{3+}, collected after different aqueous synthesis times. Silicon was used as internal standard for the quantification of the crystalline ZrO_2 fraction in the Eu^{3+}-doped samples. The Bragg reflections from Si are indicated with grey bars in Figures (a) and (b). The Bragg reflections of the monoclinic (m) and tetragonal and/or cubic (*) phases are indicated in Figure (c).

Table S1: Lattice parameters obtained from the Rietveld refinement of the Eu^{3+}- and Cm^{3+}-doped ZrO_2 samples after different aqueous synthesis times.

**Dopant concentration Synthesis time**	**Phase**	**a /Å**	**b / Å**	**c /Å**	**V / Å³**	**R_e / %**	**R_{wp} / %**
**30 ppm Cm^{3+} 16 d**	m	5.175413(1)	5.214571(1)	5.344486(1)	142.1449(6)	3.02	3.32
	t	3.61436(1)	3.61436(1)	5.224237(4)	68.24732(6)	3.02	3.32
**30 ppm Cm^{3+} 32 d**	m	5.199349(1)	5.218594(1)	5.36446(1)	143.4494(6)	2.78	3.37
	t	3.639727(2)	3.639727(2)	4.961719(2)	65.73093(3)	2.78	3.37
**30 ppm Cm^{3+} 117 d**	m	5.184404(1)	5.224184(1)	5.354001(1)	142.9363(5)	2.98	4.03
	t	3.608352(1)	3.608352(1)	5.163998(2)	67.23632(5)	2.98	4.03
**500 ppm Eu^{3+} 10 d**	m	5.056809(1)	5.276881(1)	5.20396(2)	135.3532(1)	2.71	4.52
	t	3.594693(1)	3.594693(1)	5.182119(1)	66.9624(2)	2.71	4.52
**500 ppm Eu^{3+} 12 d**	m	5.108257(2)	5.156137(3)	5.270761(3)	137.0726(1)	1.83	4.14
	t	3.601123(2)	3.601123(2)	5.167089(4)	67.00727(1)	1.83	4.14
**500 ppm Eu^{3+} 14 d**	m	5.142363(2)	5.178499(2)	5.350591(2)	140.50418(5)	1.82	3.65
	t	3.592159(2)	3.592159(2)	5.455683(7)	70.39799(5)	1.82	3.65
**500 ppm Eu^{3+} 16 d**	m	5.171081(9)	5.211321(1)	5.348873(1)	142.0484(5)	2.29	3.28
	t	3.588152(9)	3.588152(9)	5.175882(4)	66.63863(6)	2.29	3.28
**500 ppm Eu^{3+} 21 d**	m	5.16337(1)	5.211947(1)	5.349364(2)	141.9178 (7)	2.32	3.79
	t	3.569471(3)	3.569471(3)	5.207119(5)	66.34456(9)	2.32	3.79
**500 ppm Eu^{3+} 32 d**	m	5.172146(1)	5.210435(1)	5.347921(1)	142.1127(7)	2.87	5.36
	t	3.607302(2)	3.60732(2)	5.176677(3)	67.36217(6)	2.87	5.36
**500 ppm Eu^{3+} 63 d**	m	5.17491(8)	5.211042(9)	5.334986(9)	141.7894(4)	1.82	5.35
	t	3.57791(7)	3.57791(7)	5.148733(2)	65.9112(3)	1.82	5.35
**500 ppm Eu^{3+} 140 d**	m	5.169934(8)	5.211886(8)	5.354652(1)	142.19(4)	3.70	5.61
	t	3.594772(1)	3.594772(1)	5.133432(3)	66.33619(5)	3.70	5.61
**500 ppm Eu^{3+} 460 d**	m	5.177(5)	5.219102(5)	5.347019(5)	142.3695(2)	2.91	5.61
	t	3.590286(1)	3.590286(1)	5.194017(3)	66.95167(6)	291	5.61
**1 mol% Eu^{3+} 4 d**	m	5.066594 (4)	5.167452(4)	5.236912(3)	135.0869(2)	2.73	2.76
	t	3.604494(1)	3.604494(1)	5.207544(3)	67.65836(5)	2.73	2.76

**1 mol% Eu^{3+} 7 d**	m	5.0299311(4)	5.156868(3)	5.257193(2)	136.4769(1)	2.72	2.90
	t	3.588241(2)	3.5882412)	5.179887(4)	66.6935(6)	2.72	2.90
**1 mol% Eu^{3+} 8 d**	m	5.176859(7)	5.143596(7)	5.332353(6)	140.3061(3)	2.71	4.56
	t	3.640689(3)	3.640689(3)	4.972645(4)	65.91051(5)	2.71	4.56
**1 mol% Eu^{3+} 9 d**	m	5.074505(5)	5.211173(6)	5.320813(5)	139.2185(2)	2.72	3.70
	t	3.625925(9)	3.625925(9)	5.15934(2)	67.83156(2)	2.72	3.70
**1 mol% Eu^{3+} 10 d**	m	5.200087(3)	5.219239(3)	5.339239(4)	142.7911(2)	2.73	4.05
	t	3.572479(5)	3.572479(5)	5.175945(2)	66.05855(3)	2.73	4.05
**1 mol% Eu^{3+} 16 d**	m	5.169305(1)	5.21773(1)	5.35911(1)	142.0497(5)	2.79	3.33
	t	3.600959(1)	3.600959(1)	5.15415(2)	66.81088(4)	2.79	3.33
**1 mol% Eu^{3+} 32 d**	m	5.179114(9)	5.212674(1)	5.346785(9)	142.3004(5)	3.66	4.30
	t	3.608213(2)	3.608213(2)	5.199463(6)	67.69285(1)	3.66	4.30
**1 mol% Eu^{3+} 112 d**	m	5.174926(8)	5.21601(8)	5.348058(8)	142.2779(6)	3.58	5.01
	t	3.607935(1)	3.607935(1)	5.194835(2)	66.28296(5)	3.58	5.01
**1 mol% Eu^{3+} 399 d**	m	5.169488(6)	5.215702(6)	5.339124(6)	141.9474(3)	2.95	4.63
	t	3.583955(3)	3.583955(3)	5.201839(4)	66.8626(8)	2.92	4.63

STEM-EDXS

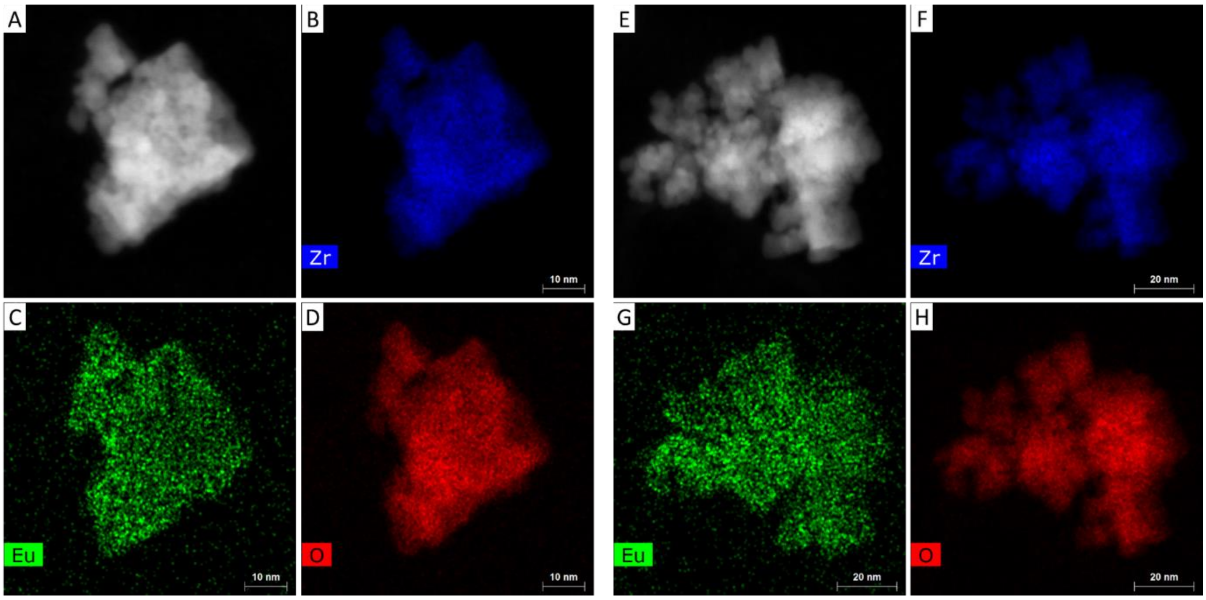

Figure S2: STEM-based analysis for the Eu:ZrO_2 samples containing 1 mol% dopant after 32 days of thermal treatment time. A and E are HAADF-STEM images, while B, C, D, and F, G, H show the corresponding EDXS-based element distribution maps for Zr (blue), Eu (green), and O (red).

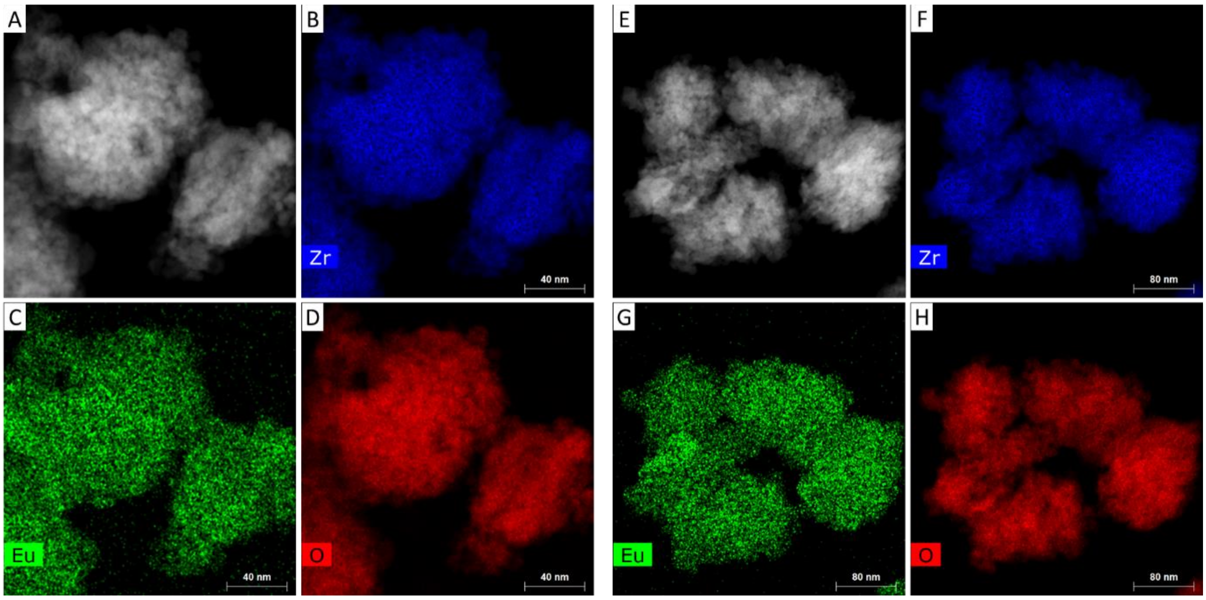

Figure S3: STEM-based analysis for the Eu:ZrO_2 samples containing 1 mol% dopant after 399 days of thermal treatment time. A and E are HAADF-STEM images, while B, C, D, and F, G, H show the corresponding EDXS-based element distribution maps for Zr (blue), Eu (green), and O (red).

Luminescence spectroscopy

Elucidating the mechanism of f-element removal from solution during the formation of hydrous zirconia

In the synthesis of the hydrous zirconia precursor material, an aqueous solution containing Zr^{4+} and the respective *f*-element was added dropwise to an alkaline NaCl solution, resulting in an instantaneous formation of a precipitate. To confirm that incorporation of the dopant cations within the hydrous zirconia structure occurs in the precipitation process, surface adsorption or the precipitation of an *f*-element-enriched secondary phase had to be ruled out. The latter experiment could only be conducted for Eu, by precipitating a hydrous Eu solid phase in the absence of Zr, *via* dropwise addition of Eu^{3+} solution into an alkaline NaCl electrolyte. The formed solid phase was recovered immediately and subjected to luminescence spectroscopic investigations. The recorded emission spectrum obtained for the Eu-precipitate is presented in Figure S4 (blue traces) together with the emission spectrum of the hydrous zirconia (co-)precipitate obtained in the presence of Eu^{3+} (black traces).

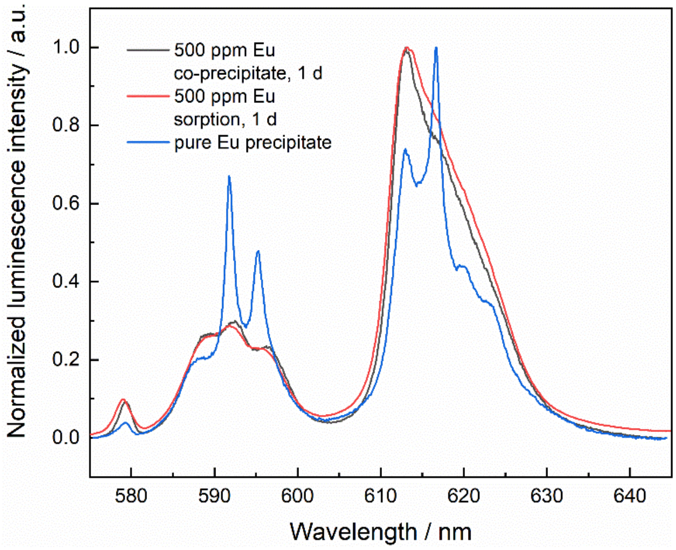

Figure S4: Emission spectra of a hydrous Eu-precipitate (blue traces), Eu^{3+}-doped hydrous zirconia (black traces), and Eu adsorbed on hydrous zirconia at pH 12 (red traces).

The emission spectrum of the europium precipitate consists of narrow emission peaks that reveal a maximum Stark splitting of both the $^7F_1$ band (585-600 nm) and the $^7F_2$ band (605-630 nm). In contrast, the hydrous zirconia co-precipitate shows very broad emission peaks without a resolved Stark splitting, pointing towards a clearly different Eu^{3+} environment within the co-precipitate. Thereby, the formation of two separate Zr-enriched and Eu-enriched hydrous precipitates in the co-precipitation step can be ruled out. The Eu-precipitate was not analyzed further, but based on the speciation diagram for europium, it is likely that it is a hydroxy carbonate phase.[1]

To account for *f*-element removal from solution via surface adsorption on the formed hydrous zirconia phase rather than co-precipitation, adsorption investigations were conducted for Eu^{3+}. In addition, Cm^{3+} sorption investigations applying luminescence spectroscopy, previously conducted by our group (Eibl et al. 2019) were compared to Cm^{3+} emission data recorded from the Cm-bearing zirconia co-precipitates

at two pH values. The experimental details are given in the materials and methods section in the main text. The Eu^{3+} emission spectrum recorded for surface-adsorbed Eu^{3+} on hydrous zirconia at pH 12 is presented in Figure S4 (red traces). The spectrum is very similar to the co-precipitate, however, with a slightly broader $^7F_0$ band (~ 578 nm) and $^7F_2$ band (605-620 nm). Due to the small differences between the luminescence emission spectra, definite conclusions of the co-precipitation mechanism, whether occurring *via* surface adsorption or incorporation, cannot be drawn from the Eu luminescence data alone.

Therefore, Cm-containing hydrous zirconia precipitates obtained at pH 5 and pH 12 were analyzed in detail. The emission spectra are shown in Figure S5 (black and red traces).

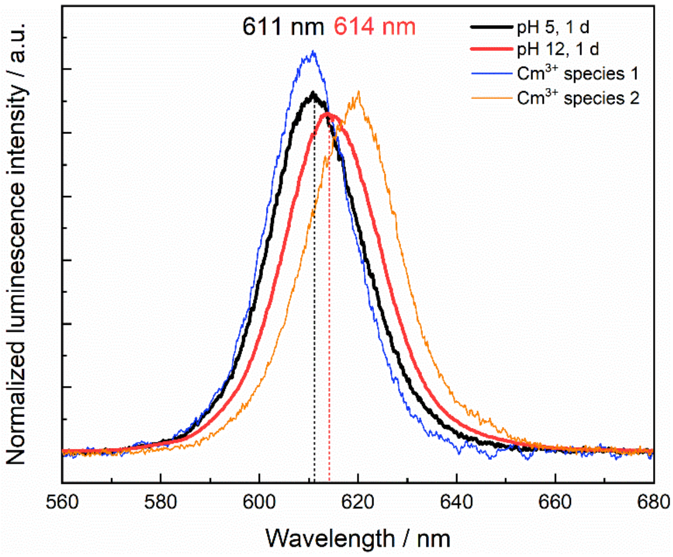

Figure S5: Emission spectra of $Cm:ZrO_2$ samples after 1 day of hydrothermal treatment time at pH 5 (black traces) and 12 (red traces) . Both spectra can be decomposed using two identical components.

The emission of the two samples show slightly shifted emission peaks at 611 and 614 nm for the sample prepared at pH 5 and pH 12, respectively. The collected spectra can be decomposed using two identical components, *i.e.* a short-lived component (species 1) and a long-lived one (species 2), Figure S5 (blue and orange traces). In other words, the Cm speciation in the hydrous zirconia precipitates differs only with respect to the relative quantities of these two components in the samples. The peak positions and the relative peak shifts collected for the samples in the two pH regions are clearly different from reported luminescence data for Cm^{3+} adsorbed on ZrO_2. Surface adsorption of Cm^{3+} at pH 5 is characterized by an emission peak at approximately 602 nm. At pH 12, surface sorption has been reported at 612 nm, *i.e.* a bathochromic shift of 10 nm occurs in the two pH regimes. Thereby, surface adsorption of trivalent *f*-elements during hydrous zirconia precipitation can be excluded. This further means, that the removal of *f*-elements from solution can only be attributed to their incorporation into the hydrous zirconia structure in the co-precipitation process.

Luminescence data following UV-excitation

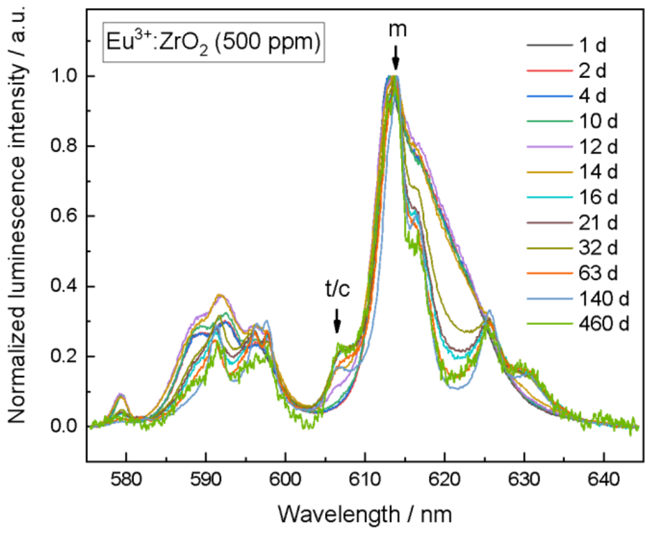

Figure S6: Luminescence emission spectra of Eu:ZrO_2 samples containing 500 ppm Eu^{3+}, collected after UV-excitation (λ_{ex} = 394 nm) at room temperature. The spectra are normalized to maximum intensity.

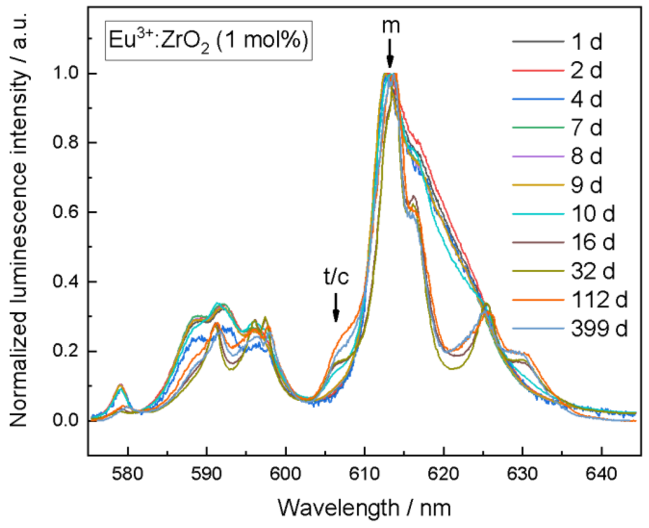

Figure S7: Luminescence emission spectra of Eu:ZrO_2 samples containing 1 mol% Eu^{3+}, collected after UV-excitation (λex = 394 nm) at room temperature. The spectra are normalized to maximum intensity.

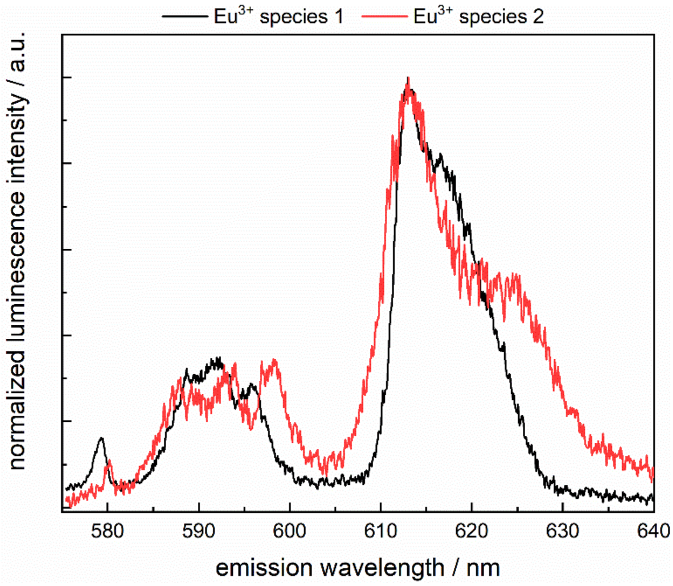

Figure S8: Two components, a short-lived (species 1) and a long-lived one (species 2), could be extracted from the recorded emission spectra of the hydrous zirconia precipitate containing Eu^{3+}. The spectra are normalized to maximum intensity.

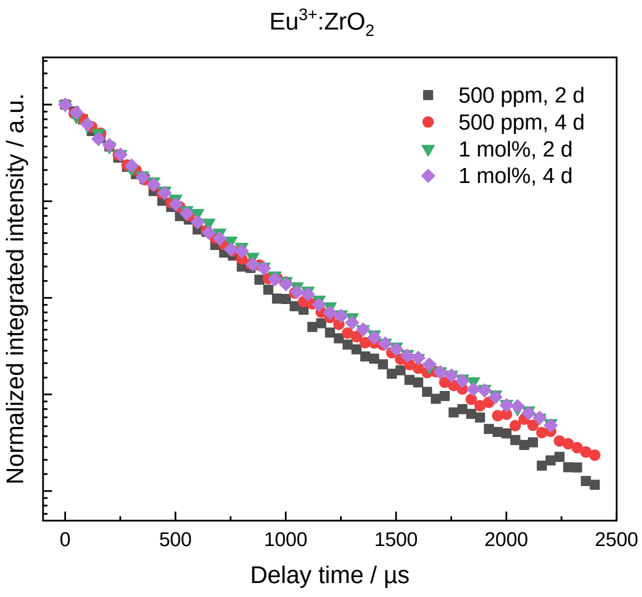

Figure S9: Selected luminescence lifetime decay curves for amorphous Eu:ZrO_2 samples with different Eu^{3+} doping concentrations and aqueous synthesis times of 2 and 4 days.

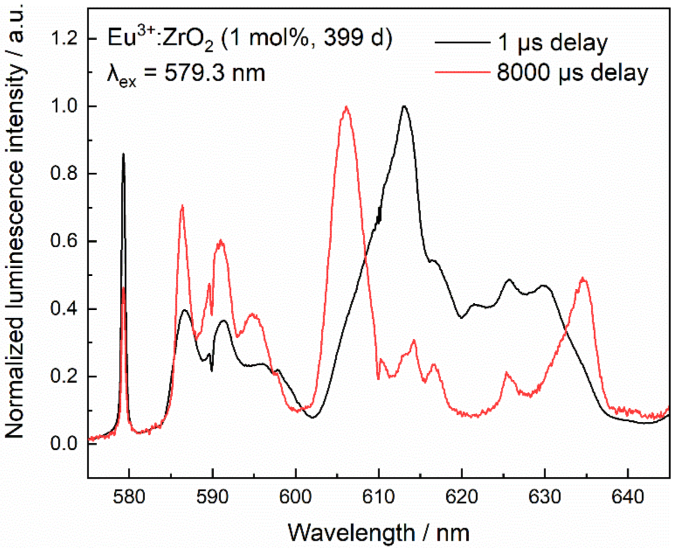

Figure S10: Emission spectra from direct excitation at different detection delay times. The spectra are normalized to maximum intensity.

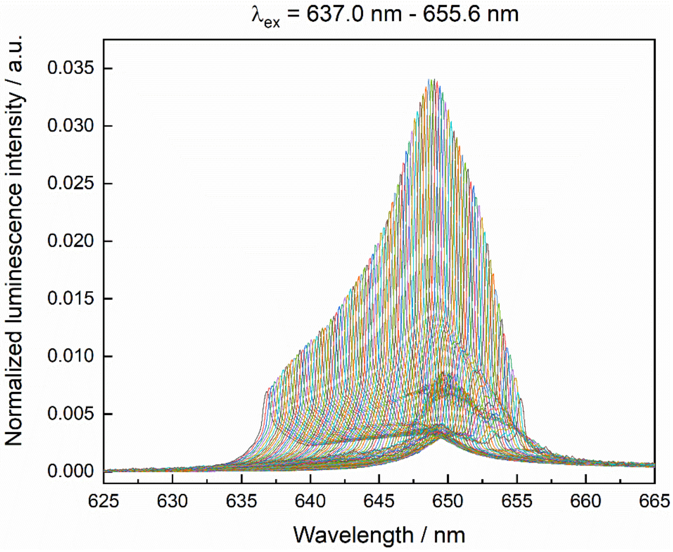

Figure S11: Cm^{3+} luminescence emission spectra of the 117-d sample, collected via excitation directly to the emitting crystal field state (A_1).

References

1. Plancque, G., Moulin, V., Toulhoat, P. & Moulin, C. Europium speciation by time-resolved laser-induced fluorescence. *Anal. Chim. Acta* **478**, 11–22 (2003).

2. Eibl, M. *et al.* A spectroscopic study of trivalent cation (Cm^{3+} and Eu^{3+}) sorption on monoclinic zirconia (ZrO_2). *Appl Surf Sci* **487**, 1316–1328 (2019).
